# What makes a compliant Phase III and pre-launch patient advocacy strategy?

**DOI:** 10.3402/jmahp.v4.33177

**Published:** 2016-12-13

**Authors:** Nick Hicks, Keith Allan, Christoph Thalheim, Paul Woods

**Affiliations:** 1 Commutateur Advocacy Communications, Paris, France; 2 Medical and Pharmaceutical Consultant, Leicester, UK; 3 European Multiple Sclerosis Platform, Brussels, Belgium; 4 Paul Woods Compliance, Stockport, UK

**Keywords:** pharmaceutical industry, patient advisory group, transparency, compliance, ethics, reputation

## Abstract

**Background:**

A key task for the pharmaceutical industry is to understand the compliance implications of engaging with a patient advocacy group (PAG). This presents challenges for the industry to negotiate the ethical and reputational issues that can arise when working with a PAG.

**Objective:**

To gain the views of pharmaceutical industry executives on future compliance challenges when working with PAGs.

**Study design:**

We conducted two surveys among two sets of industry executives: one group focussed on market access roles and the other focussed on non-market access roles.

**Results:**

Transparency was identified as the biggest challenge, followed by project rationale and then by project ownership.

**Conclusion:**

We explore how this can be overcome and make recommendations on how best to work compliantly with PAGs.

The pharmaceutical industry endeavours to understand patient experiences and address unmet patient needs by engaging with patient advocacy groups (PAGs). However, this raises important compliance, ethical and reputational considerations.

Compliance encompasses the endeavours of pharmaceutical companies to conform to relevant legislation and code of practice governing advertising, communications and interactions with customers. These have been described in a recent review (1).

In the United Kingdom, code of practice breaches are governed by the Association of the British Pharmaceutical Industry (2). Additional guidelines usually apply in the member countries of the International Federation of Pharmaceutical Manufacturers and Associations (3), which also issues universal minimum standards wherever international companies operate – even if there is no effective local regulatory framework.

This article reviews two online surveys of pharmaceutical industry executives on the compliance challenges when working with PAGs as part of a market access programme during Phase III. From these results, we present a five-part model for successfully working with a PAG in a sustainable and ethically compliant manner. To the best of our knowledge, this is the first practical discussion to make recommendations on this subject.

## Method

### Methodology

Two online surveys were conducted in December 2015 and January 2016. The first group included 35 pharmaceutical executives with varying geographical responsibility (Table 1) involved in non-market access functions (Tables 2 and 3). The second group comprised 18 executives, primarily responsible for market access (Table 2). The aim was to evaluate the groups’ understanding of the compliance considerations (Table 4) and challenges (Table 5) facing a pharmaceutical company when partnering with PAGs over the next 5 years.

**Table 1:** Geographical responsibility

What's your geographical responsibility?
National market
Regional market
European market
Global market
Established market
Emerging or developing market

**Table 2:** Commercial responsibility

Which of the following best describes your function within your organisation?
Advocacy
Compliance
Communications
Medical affairs
Policy/public/governmental affairs
Other (please specify)

**Table 3:** Main role of respondents

Survey 1 (non-market access roles)	*N*	Survey 2 (market access responsibilities)	*N*
Advocacy	6	Advocacy	1
Compliance	5	Compliance	1
Communications	6	Clinical development	1
Market access	2	Health outcomes	1
Medical affairs	11	Market access	13
Public affairs	5	Medical affairs	0
		Public affairs	0
Total	35	Total	18

**Table 4:** Compliance considerations

Rank the most important considerations for a compliant partnership project between a pharmaceutical company and a patient group
Transparency (source of funding, degree of involvement of pharmaceutical company)
Rationale for project (the primary purpose of the project)
Project implementation (primary contact, countries involved, measurement)
Project ownership (patient group, pharmaceutical company or shared ownership)
Involvement of third party (medical society or patient group medical advisory board)
Involvement of multiple sponsors
Other (please specify)

**Table 5:** Compliance challenges

In the coming 5 years, what do you foresee as the single greatest compliance challenge for a pharmaceutical company when working with patient groups?
Providing patient insight into early drug development
Ownership of patient registries
Shared access to research data from partnership projects
Markets which do not have an industry code of practice
Single sponsorship of patient groups
Involvement of patients/patient groups in HTA
Late involvement of compliance in the strategic planning process
Correct or appropriate level of funding to patient groups
Corruption
Other (please specify)

HTA, health technology assessment.

## Results

Transparency was seen as the most important common consideration for a compliant partnership (Table 6), followed by project rationale and then by project ownership (Table 7). The additional areas raised in the survey are listed in Table 8.

**Table 6:** Most important considerations for a compliant partnership project between a pharmaceutical company and a patient group (pooled results)

Most important consideration	(%)	*N*
Transparency	58	28/48
Rationale for project	35	17/48
Project ownership	23	11/48

**Table 7:** Most important considerations for a compliant partnership project between a pharmaceutical company and a patient group (by job function)

Executives with non-market access roles	Number of responses	(%)	Executives with market access responsibility	Number of responses	(%)
Transparency	25/35	71	Rationale for project	7/18	39
Rationale for project	10/35	29	Transparency	3/18	17
Project ownership	9/35	26	Project ownership/involvement of third party	2/18	11

**Table 8:** Additional areas highlighted as important

Primary job responsibility	Area identified as important
Medical affairs	Effective collaboration and credibility Publication plan and authorship
Market access	Adaptability both ways along the evolution of the project
Advocacy	Contribution of parties to project concept, for example, is the opportunity of shaping the project balanced?
Public affairs	Involvement of patient organisation from the start Publication on Internet of the project and progression Being strategically aligned to the business objectives (not a commercial business plan)
Communications	Common goal Communication towards patients around this project and towards physicians to get their buy-in

Involvement of PAGs in health technology assessment (HTA) was the most significant compliance challenge in the next 5 years, followed by appropriate level of PAG funding and then shared access to research data (Table 9).

**Table 9:** Greatest compliance challenge for pharmaceutical companies in working with patient groups (pooled results)

Greatest compliance challenge	(%)	Number of responses
Involvement of PAGs in HTA	27	13/48
Correct level of funding PAG	17	8/48
Shared access to research data	13	6/48

HTA, health technology assessment; PAG, patient advocacy group.

There was a difference between the two groups about the most important compliance challenge (Table 10). The wider group gave almost equal importance to appropriate funding of patient groups and involvement in HTA. Those working primarily in market access identified the involvement of PAGs in HTA as the priority challenge, followed by shared access to research data, with correct funding of PAGs ranked third.

**Table 10:** Greatest compliance challenge for pharmaceutical companies in working with patient groups (by job function)

Executives with non-market access roles	Number of responses	(%)	Executives with market access responsibility	Number of responses	(%)
Correct level of funding PAGs	8/35	23	Involvement of PAGs in HTA	5/13	38
Involvement of PAGs in HTA	7/35	20	Shared access to research data	2/13	16
Shared access to research data	4/35	11	Correct level of funding PAGs	1/13	8

HTA, health technology assessment; PAG, patient advocacy group.

## Discussion

### Research limitations

The principal limitations were small survey size and the multiple roles of respondents. The questions were developed with senior compliance executives, yet the possibility of response bias from question phrasing remains.

## Transparency

The approaches to compliance fall into two broad types: a legally driven approach and those founded on well-respected codes of practice.

Both laws and codes exist in all developed countries, but the balance between the two can be very different. Compliance and ethics experts should be an integral part of the team developing advocacy strategy. Ideally, executives who interact with PAGs should hold a non-commercial function, such as medical affairs or advocacy.

In disease areas where there are no PAGs, or where few pharmaceutical companies are operating, companies should beware of being a single or dominant sponsor, since this creates questions over independence. The European Federation of Pharmaceutical Industries and Associations (EFPIA) code prohibits this (4).

In emerging markets, PAGs are generally not well developed and some may not have worked with companies. Also, local teams may not be applying the same standards as international companies, and the local culture can be very different from developed countries. Work by Transparency International identifies countries with a high corruption index and can help ethical compliant relationships worldwide (5). International companies should have internal standards that ensure similar compliance and ethics requirements wherever they operate.

Best practice compliance details a company's engagement with a PAG, ideally over a number of years with an obvious research and development strategy, rather than a sales-motivated relationship, and will also include extensive record-keeping and documentation.

## Project rationale

A defined project rationale is essential to create a trust-based PAG partnership. Every interaction with a patient group should have a clearly defined legal contract which sets out why a company is working with a patient group, and what each party will gain from the partnership and in what timescale. This avoids the risk of hidden promotion.

Creating a clear rationale involves understanding the key players and their interactions, and creating an understanding in the company of a PAG's needs and perspectives. However, agreeing on a rationale means accepting and understanding the long-term nature of patient engagement. This cannot be captured in conventional sales metrics; yet there is equity value in building partnerships, which cannot be measured by traditional means.

Understanding the key influencers’ interactions shape the advocacy strategy. Thorough analysis of each advocacy group's priorities, policies and positions provides insight into the issues that matter to patients. Advocacy objectives, and therefore a project's rationale, will be different when a company creates a new partnership, compared with an established institutional presence. For example, the emphasis in a new partnership will be on understanding unmet needs and creating a mutual agenda.

## Patient group ownership

As patients are increasingly well-informed, organised and powerful, more and more companies are prioritising patient-centrism. This means integrating patient inputs in each stage of the value chain, from drug development through to commercialisation and lifecycle management. Early engagement during development – in some ways – poses fewer compliance issues, as no drug has been launched. Involvement well before the availability of a drug avoids the perception of a purely commercial interest and shows a commitment to long-term partnering with PAGs.

One of the main reasons for losing trust is companies reaching out to a PAG before there is internal clarity on what a company wants to achieve. Often, these activities are not thought through in sufficient detail in the early stages of the planning process, leading to a mismatch of shared aims.

## HTA involvement

The difference between the two groups’ views on the most significant compliance challenge illustrates an important consideration when planning an advocacy strategy; the need to think beyond partnering just for access and instead addressing unmet needs of the PAG (6, 7).

The market access executives saw the PAGs as having mainly an HTA role, whereas sustainable partnering is likely to involve working on common aims which may or may not be related to access. This was also recognised by the other responders. Internally, advocacy must therefore align with other members of the global access team to scope out priorities. How this is done depends on the way the advocacy function is built into the company infrastructure and the corporate compliance culture, and there must be a transparent, legitimate and responsible communication with patient groups, which acknowledge the sources of funding.

## PAG funding

This represents an advocacy opportunity to address unmet patient needs and a compliance challenge – especially if there are few companies in the area. Many PAGs rely on pharmaceutical funding, either as unrestricted medical grants or project funding provided for a specific activity, which must be declared by the pharmaceutical company.

Withdrawal of funding can present a PAG with a funding crisis. This is where early transparency, and a clearly defined, detailed, agreed contract, can minimise the risk of reputational damage.

## Conclusions

Pharmaceutical company interactions with PAGs must be highly regulated and transparent to avoid the risk of damage to reputation that ensues if companies fail to observe the rules and regulations set out to ensure compliance. The transparency must reinforce the legitimacy of the partnership.

This article provides a useful first step in identifying and planning how to find common points of interest to work together, by focusing on the key compliance challenges now and in the immediate future, and their solutions.

While the results need to be interpreted with caution, they provide useful insights and provoke debate on successful compliant advocacy strategy at the time of Phase III.
